# Menopause and ageing in women with multiple sclerosis

**DOI:** 10.3389/fneur.2026.1838550

**Published:** 2026-06-09

**Authors:** Cecilia Smith Simonsen, Elisabeth Gulowsen Celius

**Affiliations:** 1 Department of Neurology, Vestre Viken Hospital Trust, Drammen, Norway; 2 Department of Neurology, Oslo University Hospital, Oslo, Norway; 3 Institute of Clinical Medicine, University of Oslo, Oslo, Norway

**Keywords:** ageing, menopausal hormone therapy, menopause, MS, multiple sclerosis, reproductive ageing

## Abstract

**Background:**

The multiple sclerosis (MS) population is ageing, and a substantial proportion of women with MS are now peri- or postmenopausal. Whether menopause independently influences disease activity and progression remains unclear, and findings across studies are inconsistent. The objective of this study was to explore how menopause and biological ageing interact in MS, with a focus on inflammatory activity, disability progression, symptom burden, and clinical management.

**Methods:**

This narrative review is based on a structured literature search of PubMed, supplemented by reference screening.

**Results:**

Current evidence does not support menopause as a clear inflection point for relapse activity or MRI-defined inflammation. Similarly, recent larger studies do not demonstrate a distinct effect of menopause on Expanded Disability Status Scale (EDSS) progression after accounting for age. Emerging data suggest that reproductive ageing may be associated with increased neuroaxonal vulnerability, although findings remain limited and require replication. Symptom burden frequently worsens in midlife, reflecting overlapping effects of hormonal changes, comorbidities, and ageing, which complicates clinical interpretation and management. Evidence for menopausal hormone therapy in MS is limited; it appears to improve symptoms, but its impact on disease course remains uncertain.

**Conclusion:**

There is no convincing evidence that menopause represents an independent biological inflection point in MS. Rather, it is better understood as part of broader biological ageing, interacting with symptom burden, comorbidity, and reduced physiological reserve. A menopause-aware approach to MS care is needed to avoid misattribution and optimise management in an ageing, predominantly female population.

## 1 Introduction

Multiple sclerosis (MS) is a chronic immune-mediated disease of the central nervous system characterised by demyelination, axonal injury, and neurodegeneration (1). MS is increasingly being recognised as a disease continuum. The relative contributions of inflammation, neurodegeneration, and compensatory mechanisms vary across individuals and over time. With ageing, neural vulnerability increases and resilience declines (2). Although MS typically begins in early adulthood, a substantial proportion of people with MS are 50 years or older at the time of onset and diagnosis (3–5), and the population is ageing (6). Immunosenescence and inflammaging associated with ageing may further modify disease expression, contributing to a shift away from overt inflammatory activity toward neurodegenerative mechanisms, while also increasing susceptibility to comorbidities (7).

Women in the peri- and postmenopausal age now constitute approximately one third of the contemporary MS population (8). Menopause is defined as the permanent cessation of menstruation following the loss of ovarian follicular activity and is diagnosed retrospectively after 12 months of amenorrhoea in the absence of other causes. Definitions of menopausal status vary across studies and are frequently based on self-report. The perimenopausal transition encompasses the years of hormonal fluctuation preceding the final menstrual period. Abrupt fluctuations in oestrogen levels, such as those observed in the postpartum period (9) and around menarche (10), are associated with an increase in inflammatory disease activity in MS, and oestrogen may also exert neuroprotective effects (11). These observations have led to the hypothesis that hormonal changes during the menopausal transition may influence disease activity and progression. However, findings remain inconsistent, with uncertainty as to whether menopause constitutes an inflection point for relapse activity and disease progression (12). Nevertheless, menopausal symptoms may overlap with MS-related symptoms, and many women report worsening during the menopausal transition (13, 14). Menopausal hormone therapy (MHT) may alleviate the symptoms, but whether exogenous hormones influence MS disease progression or brain health remains uncertain (15).

Menopause in MS has emerged as a priority area across stakeholders. A recent review of two large surveys identified perimenopause and menopause as key concerns, particularly regarding their potential impact on disease activity (16). In this narrative review, we argue that menopause should be understood more as one interacting component within biological ageing, rather than a distinct inflection point, and briefly discuss current evidence and uncertainties surrounding MHT. Throughout, we highlight the inherent challenges of disentangling menopause-related effects from those of chronological ageing. Table 1 summarises the major domains discussed, together with key methodological caveats and clinical implications.

**Table 1:** Conceptual overview of menopause-related domains in midlife multiple sclerosis.

Domain	Key question	What the literature shows	Methodological caveats	Clinical implications
Inflammatory disease activity	Does menopause alter inflammatory disease activity?	Declining relapse rates after menopause, consistent with age-related reductions in inflammatory activity (12, 26).	Difficult to disentangle menopause from chronological ageing; relapse-based outcomes may lack sensitivity in later disease.	Reduced relapse rates do not equate to disease stability; conventional inflammatory measures may underestimate progression.
Disability progression	Is menopause an inflection point for disability progression?	Findings are inconsistent (12): some studies suggest accelerated progression (24, 61), while newer studies show no independent effect after adjustment for age (8, 36, 37).	Heterogeneity in menopause definitions; EDSS may be insensitive to subtle progression; inconsistent control for age and disease duration.	Menopause may represent a vulnerable window rather than a discrete inflection point; disability accrual likely reflects interacting effects of ageing, disease duration, and hormonal change.
Neurodegenerative processes	Does menopausal transition accelerate neurodegeneration?	Some longitudinal studies suggest worsening functional outcomes and structural brain measures after menopause, even after adjustment for age (36, 41).	Small sample sizes; selective outcomes; no healthy controls; limited replication; inconsistent use of biomarkers	Consistent with a model where menopause may amplify neurodegeneration already underway in midlife MS
Symptom overlap, comorbidity, and clinical ambiguity	How do menopause-related symptom changes complicate MS monitoring and management?	MS and menopausal symptoms overlap (13, 14), complicating attribution and risking misinterpretation as disease progression or treatment failure (8).	Predominantly self-reported data; attribution bias; limited longitudinal symptom tracking; lack of objective biomarkers; little data on clinical decision-making.	Symptom worsening in midlife may reflect hormonal transition, comorbidity, or ageing rather than new inflammatory activity, risking both overtreatment and undertreatment and highlighting the need for menopause-aware MS care.
Menopausal hormone therapy (MHT)	Does menopausal hormone therapy modify MS disease course or symptoms?	Very limited evidence; small feasibility trials suggest symptom benefit (57) but uncertain impact on disease course (53).	Underpowered studies; selection bias; lack of long-term outcomes; challenges with recruitment and trial design.	MHT should be considered primarily for symptom relief, not disease modification, pending stronger evidence

The table summarises key questions, principal findings, methodological caveats, and clinical implications across the domains addressed in this review.

## 2 Methods

**Figure 1:**
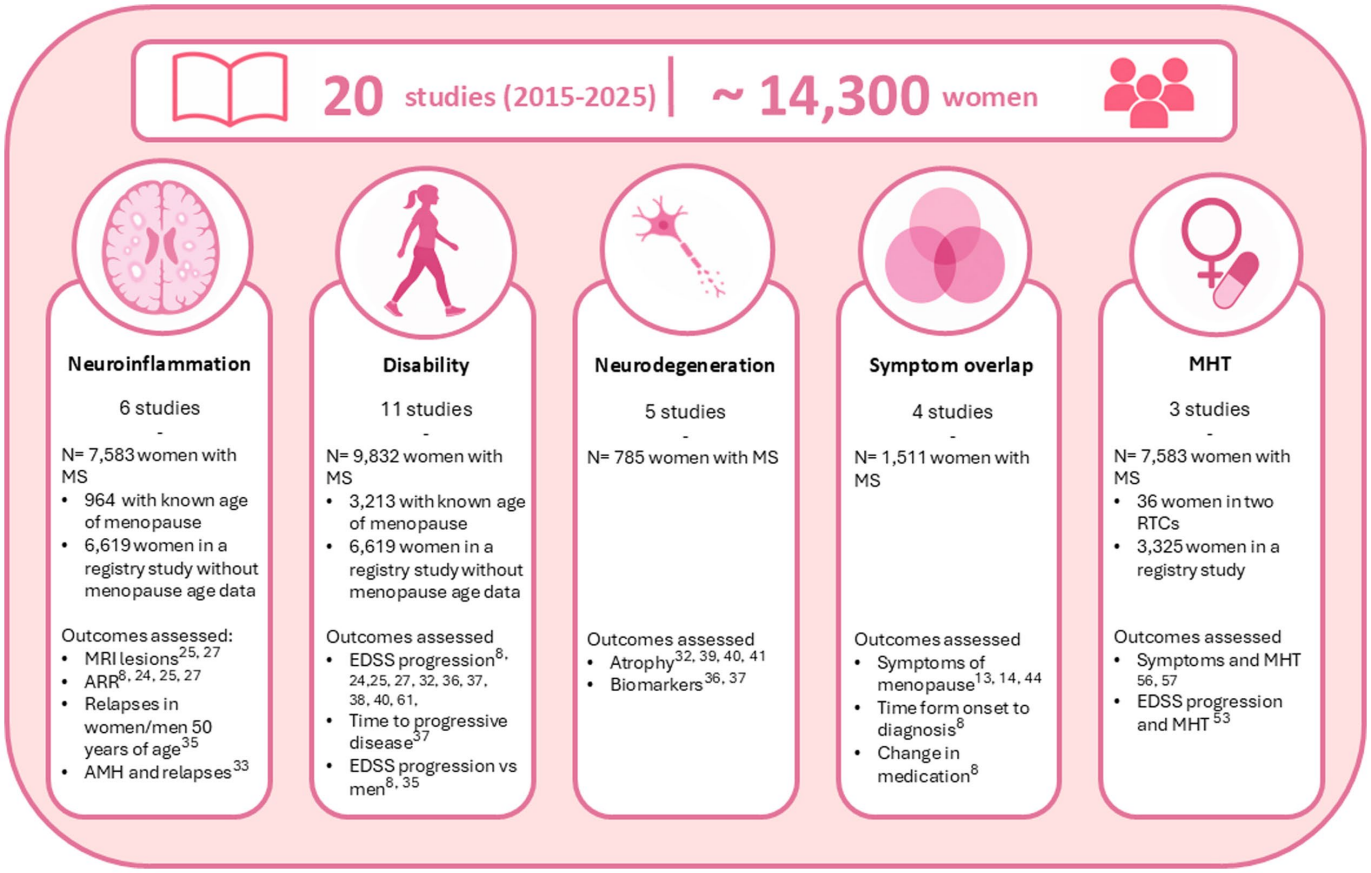
Overview of studies included in this review. MS, multiple sclerosis; MHT, menopausal hormone therapy; EDSS, expanded disability status scale; ARR, annualised relapse rate; AMH, anti-Müllerian hormone; RCT, randomised controlled trial.

This narrative review is based on a structured literature search performed in PubMed in February 2026 using combinations of the terms “multiple sclerosis,” “menopause,” “perimenopause,” “hormone replacement therapy,” “reproductive ageing,” and “ageing.” Searches were restricted to articles indexed as Clinical Study, Clinical Trial, Randomised Controlled Trial, Guideline, Meta-Analysis, or Systematic Review. No specific date limits were applied, although more recent studies and longitudinal or population-based investigations were prioritised where available. Titles and abstracts were screened for relevance, and full texts were assessed when necessary. Figure 1 illustrates the 20 clinical studies on women with MS and menopause used in this narrative review.

Additional publications were identified through reference lists of relevant articles and review papers. Given the heterogeneity in study design, menopause definitions, and outcome measures, a formal systematic review or meta-analysis was not attempted. Instead, the aim was to synthesise the available literature and highlight recurring themes, methodological limitations, and areas of uncertainty relevant to clinical practise.

**Figure 2:**
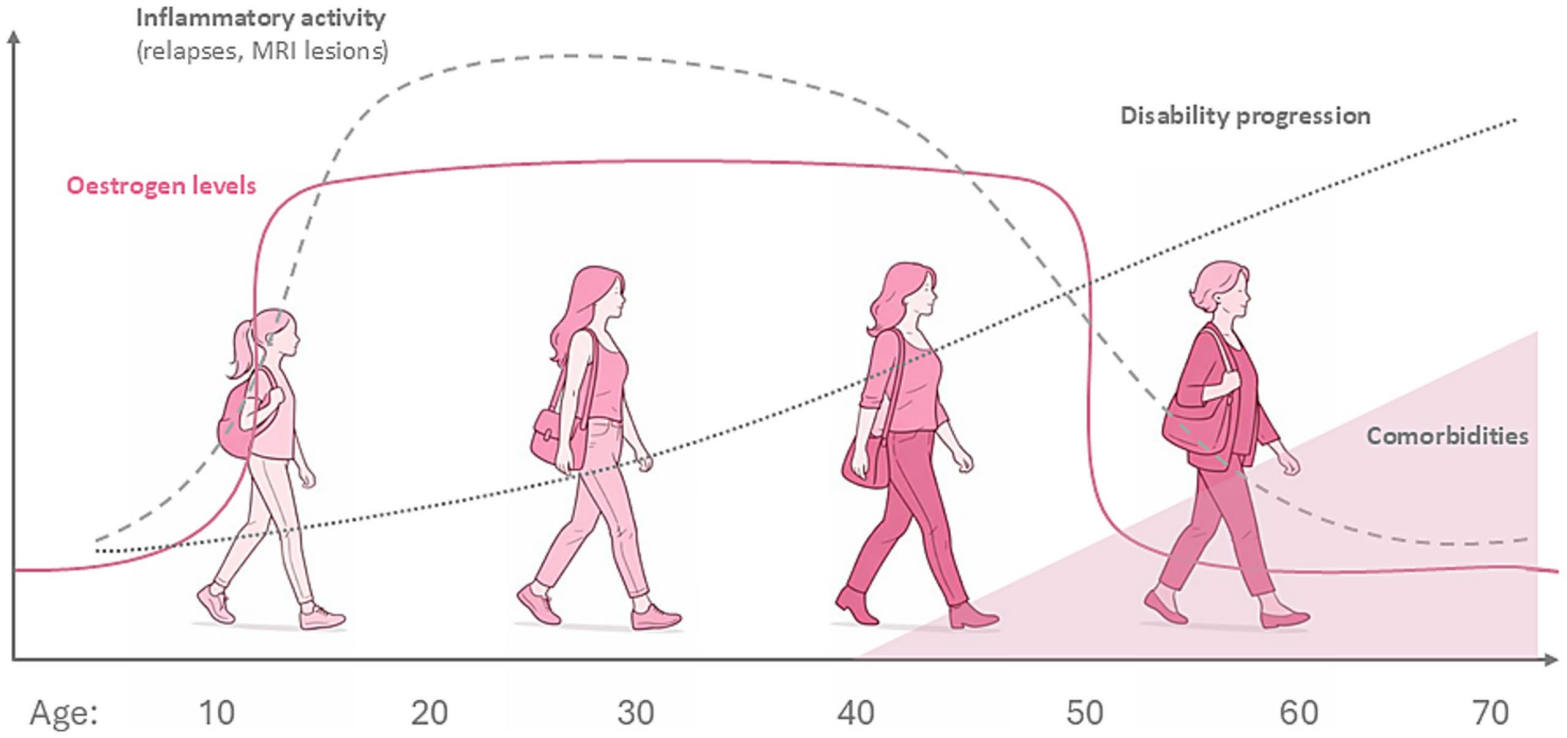
Conceptual illustration showing the interaction between ageing, oestrogen levels and disease activity and progression. The figure is conceptual and not intended to imply causality or precise temporal alignment.

Figure 2 provides a conceptual illustration of the interaction between ageing, inflammatory activity, disability progression, symptom burden, and comorbidities in women with MS. It is intended to visualise overlapping processes rather than depict quantitative relationships. Table 1 summarises the principal domains addressed in the review together with key methodological caveats and clinical implications. Table 2 shows prevalence ranges drawn from heterogeneous populations and study designs and are presented to illustrate approximate overlap rather than direct comparability.

**Table 2:** Overlapping symptom domains in menopause and MS, including reported prevalence ranges and key considerations for clinical interpretation in midlife women with MS.

Symptom	Prevalence in menopause	Prevalence in MS	Key considerations
Cognitive difficulties	40–60% (62, 63)	34–65% (64)	Cognitive symptoms in midlife women with MS may arise from declining oestrogen levels, age-related grey matter vulnerability and reduced cognitive reserve, MS-related pathology, or combined mechanisms, including sleep disruption and mood disturbance.
Depression anxiety	20–40% 25–35% (62)	30–40% 22–36% (65)	Both MS and the menopausal transition are independently associated with elevated rates of depression and anxiety; in midlife women with MS, mood symptoms may reflect inflammatory/neurodegenerative disease mechanisms, fluctuating oestrogen levels, psychosocial stressors, or combined pathways, and careful clinical contextualisation is required.
Fatigue	45–85% (66)	80% (67)	MS-related fatigue is typically multifactorial and neuroinflammatory, involving central mechanisms, lesion burden, disability level, sleep disorders, pain, mood, and medication effects. Fatigue in menopause is commonly linked to vasomotor symptoms, sleep disturbance, mood symptoms, and fluctuating oestrogen levels.
Sleep disturbances	45–55% (62, 63)	52% (68)	Sleep disturbance in midlife women with MS may reflect overlapping mechanisms, as above. MS-related nocturnal symptoms, menopausal vasomotor and hormonal changes, and shared contributors such as depression, nocturia, fatigue, and pain.
Urinary tract dysfunction	30–50% (63, 69)	65–90% (70)	Urinary symptoms in midlife may reflect neurogenic bladder dysfunction due to MS, oestrogen deficiency–related genitourinary changes, or age-related pelvic floor and comorbidity effects; careful assessment is required to avoid misattribution and delayed urological evaluation.
Sexual dysfunction	30–50% (63)	61% (71)	In MS, neurogenic and disease-related factors predominate, whereas in menopause oestrogen deficiency and genitourinary changes are central, with potential additive effects in midlife women with MS.
Heat intolerance	Hot flushes 75%	Uthoff’s phenomenon 60–80% (72)	Heat sensitivity in MS reflects temperature-dependent conduction block in demyelinated pathways, whereas menopausal hot flushes result from hypothalamic thermoregulatory instability; in midlife women with MS, vasomotor symptoms may exacerbate transient neurological worsening.

### 2.1 Women and multiple sclerosis. Why does sex matter across the MS lifespan?

Epidemiological studies consistently shows higher prevalence and incidence in women, with female-to-male ratios approaching 3:1 in many contemporary cohorts (17). The sex ratio is less pronounced before puberty (18) and appears to attenuate again in later life (4). Women typically experience earlier disease onset, and later age at menarche has been associated with a reduced risk of MS diagnosis (19). Clinically, women tend to exhibit a more inflammatory disease phenotype, with higher relapse rates and greater MRI inflammatory activity, including gadolinium-enhancing and T2 lesions, particularly in earlier disease stages (20). Relapses are often less severe and recovery more complete than in men. Women more often present with sensory symptoms and have less motor and cerebellar involvement. They have lower rates of progressive disease at onset and slower disability accumulation than men (21). They also tend to show less brain atrophy and lower rates of cognitive impairment. Finally, women have a higher lifetime risk of autoimmune disease overall. Biological sex influences immune system architecture and function, with women demonstrating stronger adaptive immune responses and heightened T- and B-cell activation, which may contribute to both increased susceptibility to autoimmune disease and a more inflammatory MS phenotype (22). These findings suggest sex-specific biological factors contribute to MS susceptibility and disease expression.

Oestrogen exerts biphasic, dose-dependent immunomodulatory effects, with lower circulating levels associated with immune activation and higher sustained concentrations associated with anti-inflammatory activity (11). In a large cohort of girls and young women with MS, relapse rates were significantly higher during the perimenarcheal period compared with pre- and post-menarche (10), supporting the concept that periods of abrupt hormonal transition may be associated with increased inflammatory disease activity in MS. This interpretation is consistent with natural history studies that demonstrate a marked increase in relapse rates in the early postpartum period (9), another phase characterised by rapid hormonal change. Beyond immunomodulation, experimental data suggest that oestrogen may exert neuroprotective effects in MS. This includes reduced microglial activation, modulation of astrocytes, preservation of blood–brain barrier integrity, and regulation of oxidative stress and apoptosis (23). Data confirming this in humans remain limited. Progesterone has also been shown to exert immunomodulatory and neuroprotective effects in experimental MS models, but the supporting evidence is more limited and less consistently demonstrated than for oestrogen (11, 21). Against this background, the menopausal transition represents a distinct midlife hormonal shift, which raises the question of how declining and fluctuating sex hormones influence inflammatory disease activity in MS. Figure 2 shows the interaction between ageing, oestrogen levels and disease activity and progression.

### 2.2 Inflammatory disease activity across midlife and menopause. Is there an inflammatory inflection point?

Inflammatory disease activity in MS generally declines with increasing age. This pattern is biologically plausible in the context of immunosenescence, as adaptive immune responsiveness declines and the capacity to mount acute inflammatory responses is reduced (7). Ageing is also accompanied by “inflammaging,” marked by chronic, low-grade innate immune activation. This may contribute to ongoing tissue injury despite fewer inflammatory events. It remains unclear whether menopause represents a true inflammatory inflection point. Age alone may explain most of the observed change (12).

While some longitudinal studies have reported a reduction in annual relapse rates after menopause (24, 25), these findings are inconsistent and appear largely driven by age rather than menopausal status per se. For example, Baroncini et al. reported a significant reduction in relapse rate following menopause, but did not observe the same effect when analyses were restricted to younger menopausal women with MS, suggesting that chronological age rather than menopausal status was the dominant factor (24). Several other studies have not identified a significant reduction in relapse rates after menopause, and a 2020 meta-analysis found no overall association between menopausal transition and relapse activity (26).

In one study, menopausal women with MS had significantly fewer new white matter and gadolinium-enhancing lesions than non-menopausal women (27), while another study found a non-significant reduction post-menopause (25). Conventional MRI inflammatory outcomes have been sparsely assessed in this context, and available data do not suggest increased neuroradiological activity after menopause. However, the evidence base is limited. Future studies should incorporate both conventional lesion measures and markers of compartmentalised or smouldering inflammation in later disease stages.

The absence of evidence for increased inflammation following the abrupt decline in oestrogen suggests that immunosenescence may already blunt acute inflammatory responses in midlife. Hormonal changes may therefore have limited capacity to translate into clinically apparent relapses or new T2 lesions. Alternatively, our current outcome measures may simply be too blunt to detect the effect. In addition, relapse rates and conventional MRI lesion measures may be relatively insensitive to later-life inflammation, particularly in individuals with longstanding disease and treatment exposure. This further complicates interpretation of menopausal effects.

### 2.3 Disability progression and neurodegeneration in midlife. If not relapses, what changes?

If menopause does not appear to precipitate an increase in inflammatory relapses, an important question is whether it instead influences neurodegenerative processes. Experimental and translational evidence indicates that oestrogen has direct neuroprotective effects in the central nervous system (CNS). This includes suppression of microglial activation, preservation of synaptic integrity, promotion of remyelination, and modulation of mitochondrial and oxidative pathways (23). Declining oestrogen levels during menopause may therefore reduce CNS resilience to injury.

Evidence from the broader literature on neurodegeneration suggests that the menopausal transition is associated with structural and metabolic brain changes, including reductions in hippocampal volume, altered white matter integrity, and reduced cerebral glucose metabolism (28). In addition, early menopause is associated with onset of several neurodegenerative diseases. Young age at surgical menopause increases cognitive decline and risk of Alzheimer pathology (29). Mortality for neurological or mental diseases is increased in women who undergo bilateral oophorectomy before the age of 45 (30). Finally, women with artificial menopause are at greater risk of developing Parkinson’s compared to women with natural menopause (31). Although these findings derive largely from non-MS populations, they provide biological plausibility that the menopausal transition may influence neuroaxonal integrity and vulnerability more broadly.

Lower levels of anti-Müllerian hormone (AMH), a marker of ovarian reserve and reproductive ageing, have been independently associated with greater disability accumulation and accelerated grey matter atrophy over 10 years in women with MS, even after adjustment for chronological age and disease duration (32). AMH levels were not associated with white matter lesion burden or T2 lesion volume in the same study. In contrast, higher AMH levels in younger women with MS have been associated with a relapse-prone phenotype (33). Together, this suggests that ovarian ageing may track more closely with neurodegeneration than with focal inflammatory activity in MS.

Women with MS onset in the peri-menopausal period appear to exhibit a phenotype that more closely resembles the male MS pattern. In a Norwegian population-based cohort, women whose disease onset occurred after menopause more frequently presented with pyramidal symptoms, had higher rates of progressive disease, and had a shorter diagnostic delay compared to women with earlier onset (8). The shorter time to diagnosis likely reflects greater functional impact at onset.

These findings align with broader observations in late-onset MS (LOMS), which is consistently associated with a more progressive course and shorter diagnostic latency. In one study, individuals with LOMS were diagnosed more rapidly than those with adult-onset MS. The authors hypothesised that increased vulnerability to cognitive and physical decline may prompt earlier clinical evaluation (34), although analyses were not stratified by sex. Data from the Danish MS registry further demonstrate that while men accrue more disability than women after the age of 45 years, EDSS progression in LOMS is similar between sexes. This suggests that age may attenuate sex-related differences in disease trajectory (35).

These observations support a model in which midlife MS increasingly converges toward a sex-neutral or “male-like” phenotype, characterised by greater pyramidal involvement and progressive disease. This pattern may reflect the convergence of age-related neurodegenerative processes and diminishing ovarian hormone–mediated neuroprotection. Rather than representing a discrete menopausal inflection point, it likely reflects the combined effects of ageing, ovarian decline, and reduced neural reserve.

Although earlier clinical studies reported conflicting findings regarding the impact of menopause on EDSS progression (12), more recent and larger cohorts have not identified menopause as a clear inflection point in EDSS disability trajectories (8, 36–38). However, this apparent stability in EDSS may obscure more subtle neurobiological changes not captured by conventional disability scales. Supporting this possibility, an MRI study demonstrated less brain atrophy in premenopausal women with MS compared to age-matched men, a difference that was no longer observed after menopause (39). Another study showed that postmenopausal women with multiple sclerosis had significantly more upper cervical spinal cord atrophy than premenopausal women, with levels comparable to men (40). In addition, Silverman et al. reported a modest but statistically significant increase in serum neurofilament light chain (NfL) levels during the menopausal transition relative to normative z-score values. This may suggest increased neuroaxonal vulnerability in this period. They also observed a slight worsening in the Timed 25-Foot Walk, whereas no significant changes were seen in the 9-Hole Peg Test, PASAT, or EDSS (36). Preliminary findings indicate that lower estradiol levels in menopausal women with MS may be associated with greater brain atrophy and higher sGFAP levels, independent of chronological age (41). However, in the absence of a contemporaneous healthy control group, it remains unclear whether these changes are specific to women with MS or reflect broader menopausal or ageing-related processes. At present, the signal is intriguing but not definitive.

The menopausal transition does not occur in isolation, but rather unfolds within the broader context of biological ageing. Immunosenescence is characterised by reduced adaptive immune responsiveness and diminished capacity to mount acute inflammatory responses, providing a biologically plausible explanation for declining relapse activity in midlife (7). At the same time, ageing is accompanied by inflammaging, marked by chronic low-grade innate immune activation and microglial priming, which may contribute to ongoing neuroaxonal injury despite fewer overt relapses. Declining oestrogen levels during menopause therefore occur within an immune milieu already shifting toward compartmentalised inflammation and reduced repair capacity. Menopause is therefore unlikely to act as a discrete trigger of progression. It may instead modify vulnerability within an already ageing immune and neural system. If neurodegenerative vulnerability in midlife is subtle and incompletely captured by conventional disability metrics, it may instead manifest primarily through symptom amplification and reduced physiological reserve.

### 2.4 Symptom overlap, comorbidities, and clinical ambiguity. Why do women feel worse?

Invisible symptoms of MS are increasingly recognised and tend to become more prominent with advancing age (42). Fatigue, cognitive complaints, sleep disturbance, mood symptoms, and urinary and sexual dysfunction are among the most common and burdensome features in later-life MS. These also overlap with the symptomatic phenotype of the menopausal transition, creating a clinical attribution problem in midlife women with MS (43). Table 2 illustrates the major areas of symptom overlap. Consistent with this, multiple studies report that many women perceive worsening of MS symptoms during peri- and postmenopause (13, 14). In addition, menopausal symptoms and MS-related symptom burden appear intertwined. One study reported significant associations between vasomotor symptoms (including hot flushes), sleep, mood and quality of life in women with MS (44). Yet menopausal health is often not addressed in routine MS care. In a survey study, most women with MS reported menopausal symptoms, but approximately two thirds had not discussed these with a healthcare provider (14). In many MS consultations, menopausal symptoms are not raised unless the patient brings them up.

Vasomotor symptoms are particularly relevant in MS because transient heat exposure can worsen neurological function via temperature-dependent conduction block (Uhthoff’s phenomenon). Hot flushes are common in midlife women, and may therefore act as a frequent, recurring amplifier of fatigue, cognitive inefficiency, gait impairment, and sensory symptoms, even in the absence of new inflammatory activity. In practise, this overlap can make clinical decisions difficult. In a cohort of 559 peri- and postmenopausal women with MS, we found that those who reached menopause before MS diagnosis experienced a 3.3-year longer diagnostic delay than those who reached menopause after diagnosis, suggesting potential misattribution of emerging neurological symptoms to menopause (8). In the same study, 30% changed, initiated, or discontinued disease-modifying therapy in the year of self-reported menopause, consistent with the possibility that menopause-related symptom change is sometimes interpreted as MS disease activity or treatment intolerance, rather than reproductive ageing (8).

Beyond symptom overlap, midlife and later life are characterised by increasing comorbidities, which further complicates symptom attribution and worsens quality of life. Importantly, comorbid conditions in midlife do not merely coexist with MS. They may interact with existing neurological impairment to amplify fatigue, gait instability, cognitive inefficiency, fracture risk, and vascular-mediated brain injury, thereby modifying overall disease expression. Menopause-related oestrogen decline accelerates bone loss, and postmenopausal women with MS have a higher osteoporosis risk than healthy age-matched controls (45, 46), with implications for falls, fracture risk, immobility and downstream disability. Cardiometabolic risk also rises after menopause, potentially compounding the already elevated vascular comorbidity burden observed in people with MS (47, 48). More broadly, immunosenescence and inflammaging increases vulnerability to infections, malignancy, frailty, and functional decline (7). These biological changes occur alongside midlife stressors, including teenage children or “empty nest” transitions, caring for ageing parents, work strain, relationship changes, and societal pressures around “ageing well.” This may magnify fatigue, sleep disturbance, mood symptoms and cognitive complaints.

Symptom worsening in midlife women with MS often reflects the combined effects of hormonal transition, accumulating comorbidity, and broader ageing processes, rather than renewed focal inflammatory activity. This clinical ambiguity increases the risk of both overtreatment (unnecessary escalation or switching of disease-modifying therapy) and undertreatment (missed menopause management, comorbidity screening, rehabilitation, or mental health support). This underscores the need for menopause-aware MS care pathways. As MS management increasingly takes a lifespan perspective, menopause-specific assessment is important to avoid confusing reproductive ageing with MS progression and comorbidities, and to better support quality of life in an ageing, predominantly female population.

### 2.5 Menopausal hormone therapy. What can we offer, and what do we not know?

Ageing cannot be halted, but the loss of oestrogen represents one potentially modifiable component of midlife biology. There is strong evidence that MHT reduces vasomotor symptoms and prevents bone loss (49). Cardiovascular effects appear to depend on timing and formulation. In the general population, potential neuroprotective effects of MHT are also thought to depend on timing, with initiation closer to menopause associated with more favourable outcomes (50). MHT’s effects on cognition in the general population are heterogeneous and continue to be debated (51). Data regarding the impact of MHT on neurodegeneration in MS remain limited (52). At present, available evidence does not support the use of MHT as a disease-modifying therapy in MS (52).

A Danish registry study of women with MS did not find an association between MHT use for less than 5 years and EDSS disability accrual, although the cohort was relatively small and other markers of neurodegeneration were not available (53). Determining whether MHT truly influences longer-term neurodegeneration or inflammation will require prospective studies that are adequately powered and carefully designed. They should incorporate clinical outcomes, cognition, neuroimaging, and fluid biomarkers. The type and formulation of oestrogen used in such trials should also be carefully considered to optimise potential neuroprotective effects while limiting risk (54).

The publication of the Women’s Health Initiative in 2002 raised significant safety concerns regarding combined oestrogen–progestin therapy (55), leading to a marked decline in MHT use, including among women with MS (53). Subsequent analyses and newer data have supported a more individualised approach, suggesting that MHT is safe when initiated appropriately in women without contraindications (49). Nevertheless, scepticism persists among both women and healthcare providers.

Concerns about safety have also contributed to slow recruitment in interventional trials of MHT in MS (15), and the number of studies in this population remains limited. A small study comparing estradiol treatment in menopausal women with MS and healthy controls reported similar improvements in hot flushes, sleep disturbance, and depressive symptoms in both groups (56). Another small trial of conjugated oestrogens plus bazedoxifene in women with MS found greater treatment satisfaction and a non-significant reduction in hot flushes, with no major safety concerns, although the sample size and treatment duration were limited (57).

Regardless of its impact on neurodegeneration, many women with MS are likely to experience meaningful improvement in menopausal symptoms that overlap with MS-related symptom burden when initiating MHT. In clinical practise, this may translate into improved sleep, reduced fatigue, and better overall functioning. In women with significant disability and reduced mobility—who may have elevated baseline thromboembolic risk—route of administration and individual risk factors warrant careful consideration, particularly given the increased venous thromboembolism risk associated with oral oestrogen formulations. In the absence of contraindications, women with MS should therefore at least be considered for MHT according to general menopause guidelines (15, 58).

Other symptomatic treatment options are available and should be considered in women with MS affected by menopausal symptoms (59), although these are beyond the scope of this review. Optimal management requires close collaboration with primary care and gynaecology, with attention to symptom management, comorbidities, bone health and cancer screening where appropriate.

## 3 Discussion

Women with MS have a different phenotype from men, and their disease trajectory and lived experience should be considered with this in mind. Menopause is a significant event in a woman’s life, representing the end of reproductive age. Given that oestrogen fluctuations have been linked to inflammatory activity in MS, one might expect menopause to alter neuroinflammation. However, available evidence does not demonstrate a consistent increase or decrease in relapse rates or conventional MRI activity at the time of menopause. A clear inflection point has not been demonstrated. This likely reflects the dominant influence of chronological ageing and immunosenescence, which attenuate acute inflammatory responses in midlife. The caveat is that most available studies rely on relapse rates and conventional MRI outcomes, and do not adequately assess compartmentalised or smouldering neuroinflammation.

Likewise, recent larger studies on MS and menopause have not demonstrated a distinct inflection point in EDSS progression at menopause. Instead, disability appears to increase gradually with age and disease duration, as observed in both women and men. Although MS trajectories also evolve with age in men, there is no direct biological equivalent to menopause. In contrast to the more abrupt decline in oestrogen at menopause, androgen levels in men decline gradually over decades (often referred to as andropause) without a clear transition point, making the clinical impact harder to define. While lower testosterone levels have been associated with greater disability and neurodegeneration in some studies, the evidence is limited (60). Future studies should examine the impact of male puberty and andropause on MS.

Menopause does not act as an abrupt accelerator of disability, although subtle neurodegenerative effects cannot be excluded. Rather, EDSS may lack sensitivity to detect more subtle or insidious changes in midlife. Emerging biomarker studies suggest that there may be modest worsening around the menopausal transition, although these signals are small and require replication. Menopause is therefore best understood as a biological modifier rather than a primary driver of MS disease progression. Loss of oestrogen may attenuate the female protective advantage, increasing vulnerability within an already ageing immune and nervous system rather than initiating new inflammatory processes. Further studies on this topic should include larger populations and healthy controls. Ideally, they should be longitudinal and include hormonal definitions of menopausal status in addition to self-defined time of menopause. Advanced neuroimaging, fluid biomarkers of neurodegeneration and glial activation, as well as patient reported outcome measures and clinical examinations should be integrated to capture changes not visible through EDSS alone. Menopausal status should be systematically addressed and corrected for in clinical trials. Key methodological priorities for future research are outlined in Table 3.

**Table 3:** Methodological priorities for future research on menopause and MS.

Domain	Key gap	Recommended study approaches
Inflammatory disease activity	Difficulty disentangling effects of menopause from chronological ageing and disease duration	Longitudinal cohorts with repeated assessment of menopausal status defined by hormonal markers and stage-specific classification, with adjustment for age and disease duration, and integration of neuroimaging, fluid biomarkers, and measures of biological ageing.
Disability progression	Limited sensitivity of conventional disability measures	Longitudinal cohorts with repeated assessment of menopausal status, multidimensional disability measures, and age-adjusted within-person trajectory analyses across the menopausal transition with adjustment for age, disease duration, and treatment exposure, ideally including comparator groups such as premenopausal women with MS and healthy female controls.
Neurodegenerative processes	Small, heterogeneous samples and limited use of appropriate control groups	Longitudinal cohorts with healthy control groups, integrating multimodal markers of neurodegeneration, including neuroimaging and fluid biomarkers (e.g., neurofilament light chain, GFAP and DNA methylation), alongside sensitive clinical measures such as cognition, upper limb function, and gait.
Symptom overlap, comorbidity, and clinical ambiguity	Difficulty disentangling MS-related symptoms from those attributable to menopause, ageing, and comorbidities	Combined use of MS-specific and menopause-specific PROMs with longitudinal follow-up across age groups, ideally including healthy controls and objective clinical measures, and accounting for comorbidity and age-related factors.
Menopausal hormone therapy (MHT)	Underpowered studies, limited long-term outcome data, and challenges in recruitment	Randomised, placebo-controlled trials with adequate sample sizes and long-term follow-up, incorporating PROMs and multimodal outcomes (including neuroimaging and fluid biomarkers) to assess both symptomatic and neurodegenerative effects, with pragmatic, patient-centred designs and embedded recruitment pathways.

GFAP, Glial Fibrillary Acidic Protein; PROMs, Patient Reported Outcome Measures.

Regardless of its impact on MS inflammation and neurodegeneration, menopause clearly impacts symptom burden and quality of life. Oestrogen decline in midlife has biological and clinical consequences that should not be overlooked in women with MS. Whether MHT can meaningfully modify neurodegeneration in MS remains uncertain, and well-designed, longitudinal placebo-controlled clinical trials are imperative. In the meantime, women with MS face a complex and cumulative disease burden, and menopause may add to this. MHT also mitigates comorbidities that are associated with MS, including osteoporosis and coronary heart disease, both of which influence long-term disability and lived experience. In the absence of contraindications, women with MS should be considered for MHT according to the same principles applied in the general population.

## 4 Conclusion

Current evidence does not support menopause as an independent inflexion point for inflammatory activity or disability progression in MS. Rather, it may function as an interacting factor within biological ageing, attenuating the female protective advantage and amplifying symptom burden. Clinically, this underscores the need to distinguish menopause-related changes from disease progression to avoid both overtreatment and undertreatment.
